# 202. The Impact and Safety of Discontinuing Routine Surveillance Blood Culture Monitoring in Allogeneic Hematopoietic Cell Transplant Recipients

**DOI:** 10.1093/ofid/ofab466.404

**Published:** 2021-12-04

**Authors:** Will Garner, Louise-Marie Oleksiuk, Elisa Malek, Kristen Reinecke, Kathleen Dorritie, Annie Im, Sawa Ito, Scott Rothenberger, Mounzer Agha, Ghady Haidar

**Affiliations:** 1 University of Pittsburgh Medical Center, Pittsburgh, Pennsylvania; 2 Hillman Cancer Center, Pittsburgh, Pennsylvania; 3 University of Pittsburgh Medical Center, University of Pittsburgh, Hillman Cancer Center, Pittsburgh, Pennsylvania; 4 University of Pittsburgh Medical Center, Hillman Cancer Center, Pittsburgh, Pennsylvania; 5 University of Pittsburgh, Pittsburgh, Pennsylvania

## Abstract

**Background:**

Bloodstream infections (BSI) cause significant morbidity and mortality after hematopoietic cell transplant recipients (HCT). Surveillance blood cultures (SBC) are commonly used to decrease the risk of developing BSI but prior data suggest limited clinical utility. At our center, SBC monitoring was discontinued on 12/1/2019. This is a single center study evaluating the impact and safety of discontinuing routine SBC monitoring.

**Methods:**

Retrospective review of allogeneic hematopoietic cell transplant recipients (HCTR) seen before (12/1/2017 – 11/30/2019) and after (12/1/2019 - 12/1/2020) discontinuation of SBC. We evaluated utility of SBC and the impact of discontinuation of SBC on admissions, mortality, and other variables.

**Results:**

One hundred thirty-six and 133 HCTR were followed before and after discontinuation of SBC, respectively. Median (range) ages were 58 (22-73) and 56 (19-73); 60 (44%) and 59 (44%) were female, respectively. The most common cancer was acute myelogenous leukemia (71 (52%) and 61 (46%)); 87 (64%) and 77 (58%) had graft-versus-host disease respectively. Pre-intervention, 1946 SBCs were drawn; 81/1946 (4.2%) were positive. Post-intervention, 29 SBC were drawn; 1/29 (3.4%) were positive. Of the 82 positive SBCs, 63 (77%) were skin flora, and 9 (11%) were gram negative rods. No cultures grew *Staphylococcus aureus* or fungi. Fifty-one (63%) of the positive SBC resulted in an admission; median (range) length of stay (LOS) was 3 days (1-11). Following discontinuation of SBC, median monthly blood culture-related admissions decreased from 3 (0-6) to 1 (0-3) shown in Figure 1. In the pre-intervention period, there were 2 BSI-related deaths, and 0 following cessation of SBCs.

Figure 1. Monthly Hospital Admissions for Positive Outpatient Blood Cultures

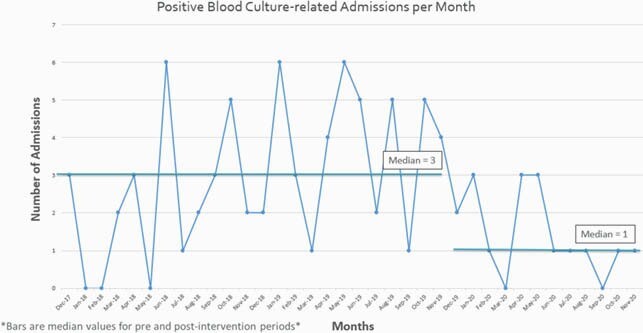

**Conclusion:**

SBCs were infrequently positive and often resulted in unnecessary antibiotic use, admission, and clinical interventions. After SBC monitoring was discontinued, there was a decrease in hospital admissions and health care utilization for positive blood cultures drawn in the outpatient setting. This intervention did not negatively impact clinical outcomes, including BSI-related mortality. Discontinuation of SBC appears to be safe and results in a reduction in healthcare utilization. Centers performing SBC should consider eliminating this practice.

**Disclosures:**

**Ghady Haidar, MD**, **Karuys** (Grant/Research Support)

